# Draft genome sequences of two *Pseudomonas* strains isolated from ^129^I plumes at the Hanford Site

**DOI:** 10.1128/MRA.00526-23

**Published:** 2023-11-10

**Authors:** Courtney J. Robinson, Jerome J. O. Oliver, Alexis Anike, M. Hope Howard, Danielle L. Saunders, Brady D. Lee

**Affiliations:** 1 Department of Biology, Howard University, Washington, DC, USA; 2 Pacific Northwest National Laboratory, Energy and Environment Directorate, Richland, Washington, USA; 3 Savannah River National Laboratory, Environmental and Legacy Management Directorate, Aiken, South Carolina, USA; Loyola University Chicago, Chicago, Illinois, USA

**Keywords:** *Pseudomonas*, bioremediation

## Abstract

*Pseudomonas* strains DVZ6 and DVZ24 were isolated from a sediment trap incubated in an ^129^I plume at the Hanford Site (Washington State, USA). Whole-genome sequencing of the strains revealed that both genomes are 5.77 Mb in size, with a G + C content of 64.75%.

## ANNOUNCEMENT

An experiment designed to culture bacteria from groundwater within a plume at the Hanford Site (Washington State, USA) containing byproducts of plutonium enrichment, including the radionuclide ^129^I, yielded multiple isolates (1). Among them were two bacteria initially identified via sequencing and analysis of 16S rRNA genes as *Pseudomonas mosselii* and designated as strains DVZ6 and DVZ24 (1). The bacteria were assessed for their potential to oxidize iodide with the goal of understanding their roles in iodine cycling at the site and future bioremediation strategies. Though both strains oxidized iodide, oxidation by strain DVZ24 was reproducibly higher than that by strain DVZ6 and several other bacteria tested (1). Here we report the whole genomes of the strains.

Strain DVZ6 was grown in Luria-Bertani broth and strain DVZ24 was grown in tryptic soy broth; both were shaken for 48 h at 28°C prior to DNA extraction with the Invitrogen PureLink Genomic DNA Mini Kit. Sequencing was performed at SeqCenter (Pittsburgh, PA). Sample libraries were generated using the Illumina DNA Prep Kit along with IDT 10 bp UDI indices and then sequenced on an Illumina NextSeq 2000, resulting in 2 × 151 bp paired-end reads. Demultiplexing, quality control, and adapter trimming were performed with bcl-convert v3.9.3 (https://emea.support.illumina.com/sequencing/sequencing_software/bcl-convert.html). The Bacterial and Viral Bioinformatics Resource Center (https://www.bv-brc.org/) genome assembly pipeline using Unicycler v0.4.8 was used to assemble reads into contigs (2, 3). CheckM was used to determine the completeness and contamination of the genomes (4). The genomes were annotated using the National Center for Biotechnology Information Prokaryotic Genome Annotation Pipeline v.6.1 (DVZ6) and v.6.4 (DVZ24) (5). The genome sequences were also uploaded to the Type (Strain) Genome Server (https://tygs.dsmz.de) for digital DNA-DNA hybridization (dDDH) analysis and species identification (6). Default parameters were used except where otherwise noted.

DVZ6 sequences were assembled into 130 contigs, and the resulting genome was 5,772,866 bp with a G + C content of 64.75%. DVZ24 sequences were assembled into 135 contigs, and the resulting genome was 5,775,344 bp with a G + C content of 64.75%. CheckM analyses indicated that the DVZ6 and DVZ24 genomes were both 99.96% complete with 0.57% contamination. Additional information regarding the genomes can be found in Table 1. Consistent with the previous study, the genomes were affiliated with *Pseudomonas* (1). However, dDDH and average nucleotide identity (ANI) analyses indicated that the previous species designation may not have been accurate. The best match based on these analyses for both strains was *Pseudomonas peradeniyensis* BW13M1 (NZ_JABWRJ020000001). However, the dDDH (formula *d_4_*) value of 62.7% (95% CI, 59.8–65.5) and an ANI of 95.69% for both strains were just below the species cut-off thresholds, yielding inconclusive results (7, 8). dDDH and ANI scores were 100% when the isolates were compared to each other; however, they reproducibly exhibit differences in iodine transformation capabilities (1) indicating a need for additional study.

**TABLE 1:** Genome information for *Pseudomonas* strains DVZ6 and DVZ24

*Pseudomonas* strain	SRA (Sequence Read Archive) accession	Genome assembly accession	Total no. of reads	Total read length (bp)	Avg. read length (bp)	N50 (bp)	Genome size (bp)	No. of contigs	Mean coverage (x)	% GC content	No. of protein coding sequences	No. of tRNAs	No. of rRNAs
DVZ6	SRX16720626	GCA_024259445.1	3,017,094	901,446,040	149	103,164	5,772,866	130	151.2	64.75	5,074	66	2
DVZ24	SRX19306820	GCA_028728275.1	9,503,527	2,462,457,984	129	107,537	5,775,344	135	420.4	64.75	5,085	66	2

## Data Availability

This Whole Genome Shotgun project (BioProject accession number PRJNA855226) was deposited in GenBank under the accession numbers JANCLM000000000 and JAQYZG000000000. The versions described in this article are JANCLM010000000.1 and JAQYZG010000000.1. Table 1 contains the SRA accession numbers.
